# Changes in the Urinary Sodium-to-Potassium Ratio Are Associated with Blood Pressure Change in Older Japanese Adults: A 7-Year Longitudinal Study

**DOI:** 10.3390/jcm11175093

**Published:** 2022-08-30

**Authors:** Takafumi Abe, Takeshi Endo, Tsuyoshi Hamano, Kenta Okuyama, Shozo Yano

**Affiliations:** 1 Center for Community-Based Healthcare Research and Education (CoHRE), Head Office for Research and Academic Information, Shimane University, Izumo 693-8501, Shimane, Japan; 2 Department of General Medicine, Okuizumo Hospital, Nita-gun 699-1511, Shimane, Japan; 3 Department of Sports Sociology and Health Sciences, Faculty of Sociology, Kyoto Sangyo University, Kyoto 603-8555, Kyoto, Japan; 4 Center for Primary Health Care Research, Department of Clinical Sciences Malmö, Lund University, 20502 Malmö, Sweden; 5 Department of Laboratory Medicine, Faculty of Medicine, Shimane University, Izumo 693-8501, Shimane, Japan

**Keywords:** salt intake, sodium-to-potassium ratio, blood pressure changes, older adults

## Abstract

Studies on the association between sodium-to-potassium (Na/K) ratio changes and blood pressure (BP) changes among older adults are limited. This 7-year longitudinal study examined the association between Na/K ratio changes (evaluated using spot urine tests) and BP changes among older Japanese adults. Data were collected from 432 participants (mean age: 70.3±4.4; range: 65–84 years) in 2012 and 2019. Changes in BP and the Na/K ratio over 7 years were calculated by subtracting baseline values from values noted during a follow-up survey. The median systolic and diastolic BP (SBP) and (DBP) changes after 7 years were 4 (IQR, −7, 14) and −1 (IQR, −9, 5) mmHg, respectively. The median Na/K ratio was changed during the follow-up period by −0.2 (IQR, −1.3, 0.7). A generalized linear model indicated that Na/K ratio changes were positively associated with SBP (B = 2.03, *p* < 0.001) and DBP (B = 0.62, *p* = 0.021) changes. In the non-antihypertensive medication-using group, urinary Na/K ratio changes were associated with SBP and DBP changes (B = 2.39, *p* = 0.001; B = 0.99, *p* = 0.033). In the antihypertensive medication user group, urinary Na/K ratio changes were associated with SBP changes (B = 1.62, *p* = 0.015). We confirmed the association between changes in the Na/K ratio and changes in BP.

## 1. Introduction

Hypertension is the second leading mortality-related risk factor causing fatal cardiovascular diseases [1]. Globally, hypertension is prevalent in 59% of women and 49% of men [2]. Approximately 46–82% of adults aged ≥60 years have hypertension in high-income countries [3]. For example, 67% of adults aged ≥65 years have hypertension in Japan [4]. Therefore, high blood pressure (BP) is still a major public health issue.

Salt intake is a well-known risk factor for increased blood pressure [5,6,7,8,9]. Major sources of sodium (e.g., soy sauce in Japan; salt in China; and bread, grains, and cereals in the United Kingdom) differ among countries [10]. Internationally, there are recommendations and campaigns to encourage reduced sodium intake. For example, the World Health Organization recommends a maximum daily salt intake of 5.0 g for adults [11]. In Japan, a salt intake of <7.5–8.0 g per day for men and <6.5–7.0 g per day for women was recommended based on dietary reference intakes for the Japanese population in 2020 by the Ministry of Health, Labor, and Welfare [12]. However, excessive salt intake remains a major global issue. Moreover, the estimation of dietary sodium intake has methodological limitations. Although the estimated 24 h sodium intake (excretion) using spot urine is a simple method, the estimated results have a low correlation with the 24 h urine collection method [13,14]. Therefore, limitations in the research methodology might lead to biased conclusions.

Recently, the 2017 American College of Cardiology/American Heart Association Hypertension Guideline recommended increasing the potassium intake and reducing the sodium intake for the prevention and treatment of hypertension [15]. Previous cross-sectional studies have shown that the sodium-to-potassium (Na/K) ratio is associated with BP [6,16], cardiovascular disease mortality, and all-cause mortality [17,18]. Potassium may reduce BP through mechanisms such as natriuretic effects and regulation of the renin-angiotensin system [19,20]. Therefore, sodium–potassium balance is an important index to consider in terms of prevention and development of hypertension. Three prospective studies, including a Japanese population study, reported that the Na/K ratio was independently associated with BP [21,22,23]. However, data regarding the longitudinal association between changes in the urinary Na/K ratio and BP changes among older adults, assessed using spot urine tests, are limited. Therefore, this 7-year longitudinal study was conducted to examine whether changes in the Na/K ratio are associated with BP changes among older adults in Japan.

## 2. Materials and Methods

### 2.1. Participants

This longitudinal study was a part of the Shimane Community-Based Healthcare Research and Education (CoHRE) study that collected data from a health examination conducted from June to October of 2012 and 2019 in Shimane, Japan. A total of 2017 adults aged ≥65 years participated in the health examination in 2012 (baseline). In total, 436 participants were also surveyed in the 2019 health examination (follow-up). After excluding four participants with missing information on study variables (Na/K ratio assessed using spot urine tests, *n* = 4), data from 432 participants were included in the longitudinal analysis. When grouped according to available or missing data, sex was not significantly different (*p* = 0.742) between the groups, whereas age (*p*  < 0.001) and body mass index (BMI; *p* = 0.047) at baseline were significantly higher among the missing data group compared with the available data group (data not shown). This study was conducted in accordance with the Declaration of Helsinki and was approved by the ethics committee of Shimane University (approval number: 2888). Written informed consent was obtained from all participants prior to study enrollment.

### 2.2. Blood Pressure

Seated BP was measured by trained nurses or public health nurses, as recommended by the Ministry of Health, Labor, and Welfare [24] and the Japanese Society of Cardiovascular Disease Prevention [25], at each community center within the study area. Systolic BP (SBP) and diastolic BP (DBP) were measured in duplicates using an automated sphygmomanometer on either the left or right upper arm after urination. The average SBP and DBP values were used in this study, and a change in BP was calculated by subtracting the baseline data from the follow-up data.

### 2.3. Urinary Na/K ratio

Sodium and potassium were assessed using spot urine tests during the health examinations. Urinary sodium and potassium were measured using a BioMajesty 6070 G analyzer (JEOL Ltd., Tokyo, Japan). The urinary Na/K ratio was calculated as sodium excreted divided by potassium excreted. Changes in the urinary Na/K ratio were calculated by subtracting the baseline data from the follow-up data.

### 2.4. Covariates

Additional data were collected in a health examination at baseline. Sex (men or women), age (years, continuous), smoking (no or yes), and alcohol consumption (no or yes) were included in this study. The use of antihypertensive medication was assessed based on the data obtained from face-to-face interviews with trained or public health nurses. The BMIs of the participants were calculated from their recorded height and weight (kg/m^2^).

### 2.5. Statistical Analyses

Descriptive statistics were calculated for all variables. The number and percentage, and the median and interquartile range (IQR), were calculated for categorical and continuous variables, respectively. Age was recorded as the mean and standard deviation (SD).

A generalized linear model was constructed to estimate the unstandardized regression coefficient (B), standard error (SE), and 95% confidence intervals (CIs) of changes in the urinary Na/K ratio for SBP or DBP changes. Analyses were performed with a crude model (nonadjusted) and a model adjusted for sex, age, BMI, smoking, alcohol consumption, and use of antihypertensive medication.

Subgroup analyses were performed to clarify whether the association between the changes in the urinary Na/K ratio and BP differed depending on the use of antihypertensive medication. A generalized linear model was also used to examine changes in the urinary Na/K ratio with a change in BP by antihypertensive medication users or nonusers. All statistical analyses were conducted using IBM SPSS Statistics 24.0 for Windows (IBM Corp., Armonk, NY, USA). This study considered *p*-values <0.05 as statistically significant.

## 3. Results

### 3.1. Participants’ Characteristics

Table 1 shows the participants’ characteristics. Of the 432 participants, 257 (59.5%) were women. The median age was 70 (IQR, 67, 74; mean, 70.3; SD, 4.4). The median urinary Na/K ratio was 2.5 (IQR, 1.7, 3.4) at baseline and 2.3 (IQR, 1.5, 3.1) at follow-up. The median urinary Na/K ratio change after 7 years was –0.2 (IQR, −1.3, 0.7). The median SBP was 128 (IQR, 116, 137) mmHg at baseline and 130 (IQR, 119, 141) mmHg at follow-up. The median SBP change after 7 years was 4 (IQR, −7, 14) mmHg. The median DBP was 78 (IQR, 72, 84) at baseline and 76 (IQR, 69, 82) at follow-up. The median DBP change after 7 years was –1 (IQR, −9, 5) mmHg.

### 3.2. The Association between Changes in The Urinary Na/K Ratio and BP

**Figure 1:**
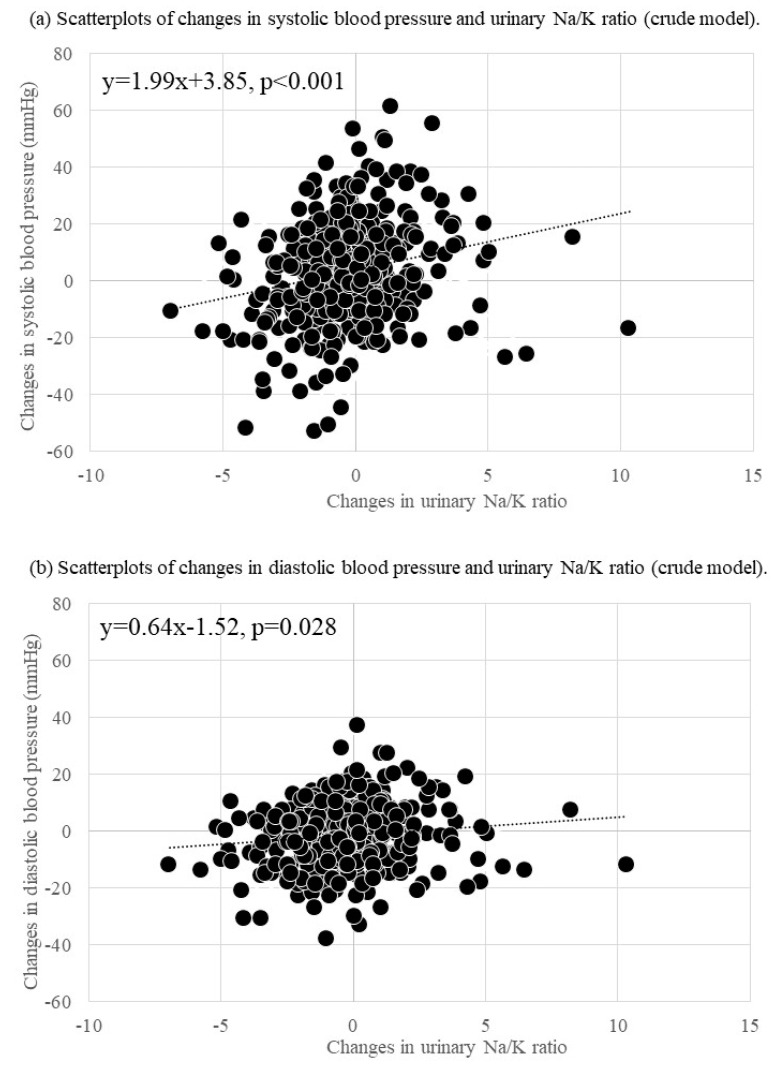
Scatterplots of changes in blood pressure and the urinary Na/K ratio.

Table 2 and Figure 1 show the associations between changes in the urinary Na/K ratio and BP change. Changes in the urinary Na/K ratio were positively associated with SBP in both the crude (B = 1.99; 95% CI = 0.93, 3.05; *p* < 0.001) and the adjusted models (B = 2.03; 95% CI = 1.04, 3.03; *p* < 0.001). A change in the urinary Na/K ratio was positively associated with DBP in both the crude (B = 0.64; 95% CI = 0.07, 1.20; *p* = 0.028) and the adjusted models (B = 0.62; 95% CI = 0.09, 1.14; *p* = 0.021).

### 3.3. Association between Changes in The Urinary Na/K Ratio and BP Resulting from The Use of Antihypertensive Medication

**Figure 2:**
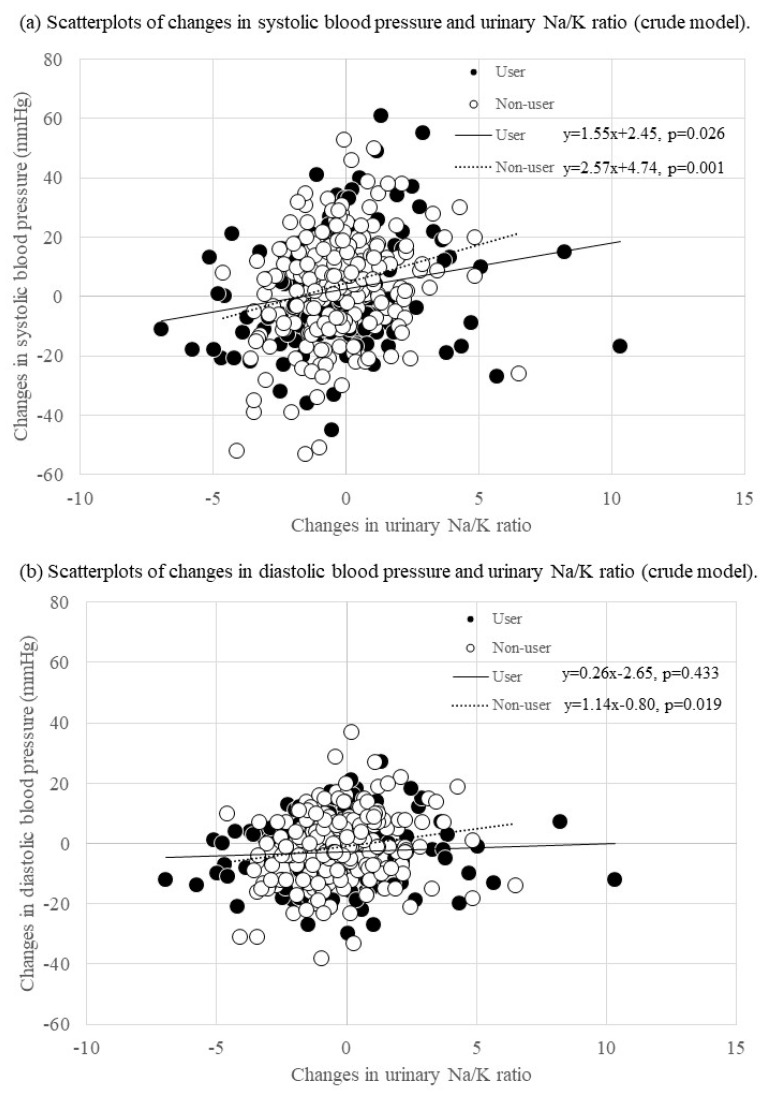
Scatterplots of changes in blood pressure and the urinary Na/K ratio by using antihypertensive medication.

Table 3 and Figure 2 show the results of the subgroup analyses. In the non-user of antihypertensive medication group, a change in the urinary Na/K ratio was positively associated with changes in SBP in both the crude (B = 2.57; 95% CI = 1.05, 4.09; *p* = 0.001) and the adjusted models (B = 2.39; 95% CI = 0.93, 3.85; *p* = 0.001) and DBP in both the crude (B = 1.14; 95% CI = 0.19, 2.08; *p* = 0.019) and the adjusted models (B = 0.99; 95% CI = 0.08, 1.89; *p* = 0.033). In the antihypertensive medication user group, although a change in the urinary Na/K ratio was positively associated with SBP change in both the crude (B = 1.55; 95% CI = 0.19, 2.92; *p* = 0.026) and the adjusted models (B = 1.62; 95% CI = 0.31, 2.92; *p* = 0.015), the DBP change showed no association in both the crude (B = 0.26; 95% CI = −0.39, 0.90; *p* = 0.433) and the adjusted models (B = 0.21; 95% CI =−0.41, 0.84; *p* = 0.504).

## 4. Discussion

To our best knowledge, this longitudinal study extends the debate regarding the changes in the urinary Na/K ratio are related to a change in BP among older Japanese adults. First, changes in the urinary Na/K ratio were independently associated with changes in SBP and DBP. Second, subgroup analyses showed that changes in the urinary Na/K ratio were associated with SBP changes in both nonusers and users of antihypertensive medication. However, the association between a change in the urinary Na/K ratio and a DBP change was only observed in nonusers of antihypertensive medication. Our main findings indicate that reducing the urinary Na/K ratio in a long-term period may be important for lowering BP. In particular, reducing the Na/K ratio is related to a reduction in SBP, regardless of the use of antihypertensive medication or not.

The results of our 7-year longitudinal study demonstrated an association between a change in the urinary Na/K ratio and changes in both SBP and DBP. These results were consistent with the findings reported by previous longitudinal studies [22,23]. A 1-year longitudinal study by Kogure et al. reported that a change in the Na/K ratio was positively associated with changes in SBP (β = 0.43, *p* < 0.01) and DBP (β = 0.22, *p* < 0.01) for the general population (mean age, 65.4 years; SD, 13.3) [23]. Higo et al. reported a positive association between Na/K and SBP (B = 1.7, *p* < 0.001) in a 5-year longitudinal study [22]. Moreover, this study showed the age-dependent association between Na/K and SBP. Namely, the Na/K ratio was correlated with a large change in SBP in older adults. Although a longitudinal study by Kieneker et al. reported that a linear association existed between the Na/K ratio and developing hypertension, after adjusting for age and sex, among Dutch populations (*p* = 0.005), there was no association between the Na/K ratio and hypertension in any of the adjusted models [21]. These findings suggested that a urinary Na/K ratio reduction over the long term might promote a reduction in BP among older adults. Therefore, future studies are required to further examine the association between a Na/K ratio reduction and a BP reduction in later life [26].

The review by Grillo et al. summarized a mechanism for the association between sodium intake and hypertension [27]; the association between high sodium intake and the increase in BP was explained by potential mechanisms, such as water retention, an increase in systemic peripheral resistance, and autonomic neuronal modulation of the cardiovascular system [27]. In contrast, the Na/K ratio is an index based on the balance of sodium and potassium intake. The consumption of vegetables and fruits, such as that promoted by the Dietary Approaches to Stop Hypertension diet, with increased magnesium, potassium, and calcium intake, has a clinically meaningful BP-lowering effect [28]. For example, potassium can aid in sodium balance and has also been demonstrated to lower BP through several mechanisms that include natriuretic effects, regulation of the renin-angiotensin system, and endothelium-dependent vascular effects [19,20]. For these reasons, our results show that changes in the Na/K ratio were associated with a BP change.

A few limitations of this study should be noted. First, as it included individuals who participated in an annual health examination across multiple centers in three towns or cities, the study sample might have resulted in selection bias. Hence, our findings cannot be applied to other populations. Second, because of the small sample size, our findings have a relatively low statistical power. In addition, participants with missing data had a slightly higher age (median, 72 vs. 70 years) and BMI (median, 22.0 vs. 22.2 kg/m^2^) than those with no missing data. Therefore, the missing data might lead to an underestimation of the association between changes in the urinary Na/K ratio and a BP change. Third, the Na/K ratio was assessed by the excretion of sodium and potassium using spot urine tests. Although the 24 h urine collection method is recommended as the gold standard for assessing sodium intake, the method is not feasible for urine collection in community settings [13]. Sodium and potassium excretion has been assessed using the spot urine test in previous epidemiological studies [6,16,17,18,21,22,23]. Recently, Tabara et al. reported the association between the urinary Na/K ratio and BP independent of clinical and environmental factors (e.g., fasting conditions) [29]. Therefore, measurement of sodium and potassium excretion using the spot urine test is considered a useful and convenient population-based method in public health practices. Fourth, our results may have been affected by regression toward the mean because of repeated measurements. Specifically, if the BP or the Na/K ratio were high at the time of the baseline survey, the values may have tended to be closer to the average values at the time of the follow-up survey. Finally, variables, including socioeconomic status, which may potentially affect the association with BP or hypertension, could not be assessed.

## 5. Conclusions

This longitudinal study determined that changes in the urinary Na/K ratio were associated with a BP change among older Japanese adults. The present findings suggest that it is necessary to reduce the urinary Na/K ratio to lower BP among older Japanese adults. Moreover, reducing the Na/K ratio is related to a reduction in SBP, regardless of the use of antihypertensive medication or not.

## Figures and Tables

**Table 1:** Participants’ characteristics (*n* = 432).

Variables	Baseline	Follow-up	7-Year Changes
Sex, *n* (%)			
Women	257 (59.5)		
Men	175 (40.5)		
Age; years old, median (IQR) Mean (SD) Minimum Maximum	70 (67, 74) 70.3 (4.4) 65 84		
BMI; kg/m^2^, median (IQR)	22.0 (19.9, 23.8)	21.9 (19.7, 23.9)	−0.2 (−1.0, 0.7)
Smoking, *n* (%)			
No	401 (92.8)		
Yes	31 (7.2)		
Alcohol consumption, *n* (%)			
No	224 (51.9)		
Yes	208 (48.1)		
Use of antihypertensive medication, n (%)			
No	275 (63.7)		
Yes	157 (36.3)		
Urinary Na/K ratio, median (IQR)	2.5 (1.7, 3.4)	2.3 (1.5, 3.1)	−0.2 (−1.3, 0.7)
SBP; mmHg, median (IQR)	128 (116, 137)	130 (119, 141)	4 (−7, 14)
DBP; mmHg, median (IQR)	78 (72, 84)	76 (69, 82)	−1 (−9, 5)

IQR—interquartile range; SD—standard deviation; BMI—body mass index; SBP—systolic blood pressure; DBP—diastolic blood pressure

**Table 2:** The association between changes in the urinary Na/K ratio and blood pressure among older adults.

	Systolic Blood Pressure				Diastolic Blood Pressure			
	B	SE	95% CI	*p*-Value	B	SE	95% CI	*p*-Value
Urinary Na/K ratio								
Crude model	1.99	0.54	(0.93, 3.05)	**<0.001**	0.64	0.29	(0.07, 1.20)	**0.028**
Adjusted model †	2.03	0.51	(1.04, 3.03)	**<0.001**	0.62	0.27	(0.09, 1.14)	**0.021**

B—unstandardized regression coefficients; SE—standard error; CI—confidence interval † The urinary Na/K ratio was examined separately. Sex, age, body mass index, smoking, alcohol consumption, and use of antihypertensive medication were adjusted. Values in boldface indicate significance (*p* < 0.05).

**Table 3:** Subgroup analyses of the association between changes in the urinary Na/K ratio and blood pressure using antihypertensive medication.

			Systolic Blood Pressure				Diastolic Blood Pressure			
			B	SE	95% CI	*p*-Value	B	SE	95% CI	*p*-Value
Antihypertensive medication										
	Nonuser									
	Urinary Na/K ratio	Crude model	2.57	0.78	(1.05, 4.09)	**0.001**	1.14	0.48	(0.19, 2.08)	**0.019**
		Adjusted model	2.39	0.74	(0.93, 3.85)	**0.001**	0.99	0.46	(0.08, 1.89)	**0.033**
	User									
	Urinary Na/K ratio	Crude model	1.55	0.70	(0.19, 2.92)	**0.026**	0.26	0.33	(−0.39, 0.90)	0.433
		Adjusted model	1.62	0.67	(0.31, 2.92)	**0.015**	0.21	0.32	(−0.41, 0.84)	0.504

B: unstandardized regression coefficients; SE: standard error; CI: confidence interval The urinary Na/K ratio was examined separately. Sex, age, body mass index, smoking, and alcohol consumption were adjusted. Values in boldface indicate significance (*p* < 0.05).

## Data Availability

Data are available upon reasonable request. This study used data from the Shimane CoHRE (Center for Community-Based Healthcare Research and Education) study. Some of the data are available from the CoHRE, Organization for Research and Academic Information, Shimane University, 223-8 Enya-cho, Izumo-shi, Shimane 693-8501, Japan.
